# Exploration of the distribution and host range of *Phloeosinus deleoni* Blackman (Curculionidae, Scolytinae) through collection records, field collections and modelling of potential distribution

**DOI:** 10.3897/BDJ.13.e164908

**Published:** 2025-10-01

**Authors:** Montserrat Cervantes-Espinoza, Jose Luis Maya-Ramos, Thomas Atkinson, Enrico Alejandro Ruíz, Francisco Armendariz-Toledano

**Affiliations:** 1 Laboratorio de Ecología, Departamento de Zoología, Escuela Nacional de Ciencias Biológicas, Instituto Politécnico Nacional, Ciudad de Mexico, Mexico Laboratorio de Ecología, Departamento de Zoología, Escuela Nacional de Ciencias Biológicas, Instituto Politécnico Nacional Ciudad de Mexico Mexico; 2 Colección Nacional de Insectos, Departamento de Zoología, Instituto de Biología, Universidad Nacional Autónoma de México,, Ciudad de Mexico, Mexico Colección Nacional de Insectos, Departamento de Zoología, Instituto de Biología, Universidad Nacional Autónoma de México, Ciudad de Mexico Mexico; 3 Facultad de Estudios Superiores Iztacala, Universidad Nacional Autónoma de México, Ciudad de Mexico, Mexico Facultad de Estudios Superiores Iztacala, Universidad Nacional Autónoma de México Ciudad de Mexico Mexico; 4 University of Texas, Texas, United States of America University of Texas Texas United States of America; 5 Laboratorio de Ecología, Departamento de Zoología, Escuela Nacional de Ciencias Biológicas, Instituto Politécnico Nacional., Ciudad de Mexico, Mexico Laboratorio de Ecología, Departamento de Zoología, Escuela Nacional de Ciencias Biológicas, Instituto Politécnico Nacional. Ciudad de Mexico Mexico

**Keywords:** bark beetles, *
Juniperus
*, distribution pattern, environmental variables, biogeographic provinces, host

## Abstract

*Phloeosinus deleoni* Blackman, 1942 (Coleoptera, Curculionidae, Scolytinae) is a bark beetle for which limited information exists regarding its ecology, distribution and host range. The objective of this study was to delimit its current distribution, identify confirmed host plants, explore possible additional hosts and model its potential distribution, based on the availability of known hosts. Records from scientific collections and field sampling were used to define its current distribution and Maxent in RStudio was applied to generate potential distribution models. New records were obtained in Durango, although the general distribution pattern remained congruent with previous knowledge. *Juniperus flaccida* and *Juniperus deppeana* were confirmed as primary hosts, with direct feeding observed on *J. deppeana*, expanding its known host range. While associations with other *Juniperus* species were considered, available data and distribution maps did not support this hypothesis. Environmental variables, particularly the temperature of the warmest month and annual precipitation, accounted for a substantial portion of the variation in ecological suitability. However, the presence of climatically suitable areas lacking records of occurrence indicates potential regions for colonisation. The absence of records in these areas is likely related to host ecology, as well as biological, dispersal and geographical constraints of *P. deleoni*. Potential distribution models indicated high environmental suitability in the biogeographic provinces of the Sierra Madre del Sur, Sierra Madre Oriental and the Transmexican Volcanic Belt. Overall, the results reinforce the hypothesis that *P. deleoni* is an oligophagous beetle, closely related to *Juniperus* species, with a host-use bias towards *J. flaccida* and is considered a native species restricted mainly to the central region of the Mexican Transition Zone.

## Introduction

Bark beetles of the genus *Phloeosinus* Chapuis, 1869 (Coleoptera, Curculionidae, Scolytinae) are phloeophagous, feeding on the phloem, a tissue of the vascular system of trees responsible for nutrient transport (Wood 1982). They primarily colonise the branches and trunks of felled or dead trees, although some species can also infest living trees weakened by disease or drought (Moraal 2010, Lindgren and Raffa 2013). The genus is widely distributed across North America, Europe, Asia, North Africa and Australia (Wood 1982, Wood 1986). Currently, *Phloeosinus* comprises approximately 80 described species, of which at least 66 are taxonomically valid. These species are mainly distributed in the Nearctic and Palaearctic Regions (Faccoli and Sidoti 2013, Kirkendall et al. 2015). Nearly 50% of the species, including 26 species and four subspecies, occur in North and Central America, particularly in temperate and subtropical regions (Wood 1986, Bright and Skidmore 1997, Atkinson 2022). While *Phloeosinus* species worldwide exhibit a preference for conifers, the American species have specialised in feeding and reproducing on members of the family Cupressaceae, including *Cupressus* Linnaeus, 1753; *Chamaecyparis* Spach, 1841; *Thuja* Linnaeus, 1753; *Juniperus* Linnaeus, 1753; *Hesperocyparis* Bartel and R.A., 2014; and *Taxodium* Rich, 1810 (Adams et al. 2009, Hernández-García et al. 2020, Cervantes-Espinoza 2022).

Amongst the species of *Phloeosinus*, *Phloeosinus deleoni* Blackman, 1942, exhibits a patchy distribution across the temperate, arid and semi-arid regions of Mexico. Geographic records indicate its presence in four major mountain systems: the Sierra Madre Occidental (Chihuahua, Durango), the Transmexican Volcanic Belt (Hidalgo, Michoacán), the Sierra Madre del Sur (Oaxaca, Puebla) and the Sierra Madre Oriental (Nuevo León) (Wood 1982). This species is considered an early successional saprophyte, colonising and decomposing phloem from dead, freshly cut or dying plant material, in particular, branches and trunks of *Juniperus* spp. (Wood 1982). Through this process, *P. deleoni* plays an essential role in nutrient cycling in xeric habitats (Atkinson and Equihua 1985, Atkinson and Equihua-Martínez 1986, [Bibr B12914417][Bibr B12914722]).

Despite its ecological importance, the distribution of *P. deleoni* remains poorly studied. Most records are based on old and sporadic collection events, typically reported using state or national divisions and lacking precise locality data (Wood 1986, Atkinson et al. 1986, Atkinson and Equihua-Martínez 1986, Adams et al. 1990, Nápoles et al. 1997). Moreover, its host range has yet to be thoroughly verified. In contrast, *P. deleoni* has been associated with *Juniperus* spp., *Juniperus deppeana* Steud and *Juniperus flaccida* Schltdl, with confirmed and repeated records only for the latter (Wood 1982, Wood 2007). Evaluating these host associations is essential, as identifying the specific *Juniperus* species with which *P. deleoni* interacts may reveal whether the beetle's distribution is linked to the geographic availability of its hosts. Another key aspect of *P. deleoni* ecology that remains unknown is the role of environmental factors in shaping its distribution and whether its geographic range coincides with that of its host plants. This lack of information hinders the understanding of its ecological interactions and limits the development of effective management and conservation strategies for this species (Cognato et al. 2021).

*Phloeosinus deleoni* was initially described from Jacala, Hidalgo, based on specimens collected from *Juniperus flaccida* (Blackman 1942). Since its description, approximately half of the ecological records available from entomological collections for this species have been associated with *J. flaccida*; some records list *Juniperus* spp. and *J. deppeana* as hosts. However, verifiable documentation for *J. deppeana* is lacking, so this association remains uncertain (Wood 2007, Atkinson 2024). The most frequently recorded host, *J. flaccida*, is commonly known as juniper, cedar, red cedar, drooping-leaf juniper or sabino. It can grow up to 1 m in diameter and reach heights of 20 m (Aguilera 2001). *Juniperus flaccida* has a broad latitudinal distribution and is considered native to Mexico (Atkinson and Equihua 1985, Aguilera 2001). To date, all known records of this beetle have been reported exclusively from Mexico. In the past 20 years, the host has undergone nomenclatural changes. Today, the concept of *J. flaccida* includes at least three species of flexible-leaved junipers from Mexico: *J. flaccida*, J. poblana (Martínez) R. P. Adams (formerly J. flaccida var. poblana Martínez) and *J. martinezii* Pérez de la Rosa (Pérez de la Rosa 1985, Adams 2014). The relationship between *J. flaccida* and *J. poblana* is complex and taxonomically challenging due to their morphological similarity and ability to hybridise (Zanoni and Adams 1979, Adams and Schwarzbach 2015); however, recent phylogenomic analyses suggest that *J. flaccida* and *J. poblana* may be sister species, but the statistical support for this relationship is weak (Adams et al. 2018).

The habitat of the three flexible-leaved juniper species is very similar to that of *P. deleoni*; they are primarily established in arid and semi-arid climates, at altitudes ranging from 1,200 to 2,440 m a.s.l., forming small vegetation patches in rocky areas, slopes and mixed forests (Miranda and Hernández-X 1963). These junipers occur in transition zones between *Quercus* Linnaeus, 1753, *Pinus* Linnaeus, 1753 and *Abies* Mill, 1754 forests, as well as xerophilous scrub, grasslands or, occasionally, tropical deciduous forest (Zamudio and Carranza 1994, Yanes et al. 2001). In addition, these ecosystems are found in mountain ranges within the Mexican transition zone, which marks the boundary between the Neotropical and Nearctic biogeographic regions (Adams et al. 1990, Morrone 2020) and have confirmed records in 22 Mexican states (Martínez 1963, Zanoni and Adams 1979, Adams et al. 1990, Adams et al. 2009, Gernandt and Pérez-de la Rosa 2014). In the United States, the flexible-leaved juniper species has been documented in southwestern states such as California, New Mexico, and Arizona, and in the southern state of Texas (Zanoni and Adams 1979, GBIF 2024a).

Flaccid‐leaved *Juniperus* species are ecologically significant due to their resilience to harsh climatic and environmental conditions, particularly in xeric and semi-arid environments. Their ability to withstand degradation allows them to maintain essential ecosystem functions, such as stabilising habitats, preventing soil erosion and supporting nutrient cycling in these challenging ecosystems (De Aizpúrua 1995). However, selective and intensive timber extraction has altered their population structure, resulting in reduced densities of large-diameter trees (Ayerde-Lozada and López-Mata 2016), even though the area occupied by *J. flaccida* exceeds 20,000 km² (Borja de la Rosa et al. 2010, Ayerde-Lozada and López-Mata 2016). *Juniperus poblana* is classified as near-threatened and *J. martinezii* as vulnerable, mainly due to the impacts of recurrent fires and overgrazing, which compromise their habitats in xeric and semi-arid regions (Farjon 2000, Farjon 2011, Farjon 2013, IUCN 2025). Consequently, these threats, along with those affecting *J. flaccida*, highlight the vulnerability of these species and underscore their importance for sustaining ecosystem functionality in dryland environments (Farjon 2000, Farjon 2011).

*Phloeosinus deleoni* is one of the bark beetle species distributed in Mexico that combines a wide geographic range with a close ecological association with *Juniperus*, one of the most widespread tree genera in the country, occurring in both temperate and xeric environments. Although it has been reported as monophagous on *Juniperus flaccida* (Fernández-Campos et al. 2025), detailed reviews of biological collections, digital repositories and specialised literature suggest that it may also be associated with other *Juniperus* species, thereby expanding its known host range. Despite its ecological importance in nutrient cycling and its potential as an indicator of ecosystem health in juniper woodlands, its distribution, host associations and environmental preferences remain poorly documented (Atkinson and Equihua-Martínez 1986, Moraal 2010, Cervantes-Espinoza 2022, Fernández-Campos et al. 2025). This lack of up-to-date knowledge limits our understanding of the ecological role and biogeographic patterns of *P. deleoni*, highlighting the need for targeted studies that integrate distribution data, host verification and environmental factors.

The ecological association between *P. deleoni* and *Juniperus* species suggests that this bark beetle may have a broader host range and a wider distribution than what is currently documented. Several factors contribute to this hypothesis: (1) the habitat similarity between *P. deleoni* and multiple *Juniperus* species, particularly in xeric and semi-arid regions; (2) the lack of precise host identification in many of the beetle's recorded occurrences; (3) taxonomic uncertainties surrounding some *Juniperus* species; and (4) the possibility that *P. deleoni* is capable of utilising a more extensive range of host species than previously recognised. This study aimed to determine the current and potential distribution of *Phloeosinus deleoni* in Mexico, using the availability of *Juniperus* species reported as its hosts as a key factor influencing its presence. Additionally, the study sought to evaluate the associations between *P. deleoni* and its host plants, based on host records from specimen labels and updated field verifications. A detailed analysis of environmental variables was also conducted to identify which factors play the most significant role in shaping the distribution of *P. deleoni* across Mexico.

## Material and methods

Occurrence data for *Phloeosinus deleoni* were obtained from the bark and ambrosia beetle database (Atkinson 2022, https://www.barkbeetles.info) and from eight entomological collections: Colección de Insectos Universidad Autónoma de Nuevo León, Linares (UANL); Comisión Forestal del Estado de Michoacán, Morelia (COFOM); Colección Científica de Entomología Forestal, División de Ciencias Forestales, Universidad Autónoma Chapingo (UACH); Colección Entomológica del Instituto de Fitosanidad, Colegio de Postgraduados en Ciencias Agrícolas, Campus Montecillo (CEAM); Universidad Autónoma del Estado de México, Estado de México (UAEM); Colección Nacional de Insectos, Instituto de Biología Universidad Nacional Autónoma de México (CNIN); La Colección Entomológica de la Escuela Nacional de Ciencias Biológicas, Instituto Politécnico Nacional (ENCB), Laboratorio de Análisis de Referencia en Sanidad Forestal de la Secretaría de Medio Ambiente y Recursos Naturales, Coyoacán, Mexico; and Smithsonian Institution, National Museum of Natural History (USNM), USA. Field expeditions were conducted at multiple sites within the species' range, including northern (Nuevo León) and southern (Oaxaca) Mexico, which were included in this study. At each site, *Juniperus* trees were identified in the field using keys from Schulz et al. (2005) and cross-checked with recent taxonomic literature. To account for the recent division of *J. flaccida* into three sympatric species (*J. flaccida, J. poblana* and *J. martinezii*), host identity for previously collected specimens was carefully verified using specimen labels and updated taxonomic references. Recently collected beetle individuals, including both males and females, were examined, identified and deposited in the CNIN entomological collection. All collecting activities were conducted under permits issued by the Secretaría del Medio Ambiente y Recursos Naturales of Mexico (FAUT-0352, FAUT-0353).

Specimens from field expeditions and those from entomological collections were identified, based on morphological characteristics of the head, pronotum, abdomen and the sculpture of the elytral declivity, following the diagnostic criteria established by Wood (1982). To confirm taxonomic identity, a detailed comparison was made with the original species description published by the same author (Wood 2007) and with the characters established by Fernández-Campos et al. (2025). High-resolution images of a representative specimen were captured using a ZEISS Axio Zoom V16 microscope, equipped with an Axiocam 305 colour camera and processed using Zen 3.5 Blue Edition software.

Occurrence data for hosts *Juniperus flaccida* (173 records), *J. martinezii* (78 records), *J. poblana* (77 records) and *J. deppeana* (793 records) were obtained from the Global Biodiversity Information Facility (GBIF 2023, GBIF 2024a, GBIF 2024b, GBIF 2025). A total of 1,600 records were retrieved from GBIF. After the data cleaning process, 1,121 records were retained for analysis. The data cleaning process involved removing duplicate records from the same locality with identical dates, excluding records without precise geographic coordinates and selecting only those associated with voucher specimens deposited in a biological collection. This latter criterion is relevant because these records are tied to actual specimens with verifiable data, ensuring the reliability and taxonomic accuracy of the information. Records based solely on human observations were excluded, as they cannot be independently confirmed and may introduce errors in host or distributional data. The final dataset included only records that provided reliable information on the host's locality. To visualise the spatial distribution of each species, occurrence records were projected using ArcMap ver. 10.8 (ESRI 2020). Since state boundaries can introduce artificial delineations, the current and potential distributions of bark beetles were analysed within the framework of the biogeographic provinces of Mexico proposed by Morrone (2019). These provinces reflect biodiversity patterns shaped by evolutionary history, environmental factors and geological events. However, distribution based on Mexico’s state boundaries was also included for practical purposes and for those unfamiliar with biogeographic provinces.

The calibration area for the *P. deleoni* distribution model was determined, based on the distribution of its host plants. A buffer of 40 km was applied around each occurrence point to estimate potential dispersal range within *Juniperus* populations (Ayerde-Lozada and López-Mata 2016). The buffer included four *Juniperus* species recognised as hosts: *J. flaccida*, *J. martinezii*, *J. poblana* and *J. deppeana*. These taxa not only co-occur in similar environments, but were historically treated as varieties of *J. flaccida*. Only after the taxonomic revision by Adams et al. (2018) were they recognised as distinct species, so many older records do not differentiate amongst them. This buffer was biologically justified using flight capacity estimates of bark beetles from the genus *Dendroctonus* Erichson, 1836 (Chen et al. 2010, Evenden et al. 2014), which are ecologically similar and inhabit montane coniferous forests. Reported dispersal distances range up to 0.27 km in *Dendroctonus armandi* Tsai and Li, 1979 and forest fragment distances of up to 24 km still allow dispersal in *Dendroctonus ponderosae* Hopkins, 1902 (Chen et al. 2010, Evenden et al. 2014). Despite being smaller, *P. deleoni* is expected to have lower dispersal capacity, making 40 km a conservative yet biologically realistic buffer. This value also aligns with the spatial distribution of *Juniperus flaccida* forests, which are often separated by 10–45 km, ensuring ecological realism in modelled areas (Ayerde-Lozada and López-Mata 2016).

A shapefile was generated from the buffer and used as the background area for ecological niche modelling. Biogeographic provinces (Rojas‐Soto et al. 2024) were used as complementary references to describe spatial distribution patterns of *P. deleoni*. Most provinces are included in the buffered area, except for parts of the Balsas Basin, where *Juniperus* is scarce.

To distinguish the possible differences between the altitudinal ranges of *P. deleoni* and its hosts, a violin plot showing the distribution of altitudes for *P. deleoni* and its probable hosts was made, accompanied by a boxplot that provides additional information on the median and quartiles of the distribution. The analysis was performed in ggplot2 (Wickham 2016) for RStudio (Rstudio 2021).

To analyse the environmental factors influencing the distribution and potential distribution of *Phloeosinus deleoni*, 19 bioclimatic variables were obtained from the WorldClim database (Fick and Hijmans 2017) at a 30 s (~ 1 km) resolution. The satisfactory resolution was chosen because bark beetles are a small species and such a resolution is necessary to capture the fine-scale environmental conditions related to their endophytic habits. All variables were extracted for each insect occurrence record and a normality test was performed. To address multicollinearity, a Pearson correlation test was conducted and only variables with a correlation coefficient r < 0.8 were retained. From these, the most ecologically relevant variables for the species distribution model were selected using a Jackknife test in Maxent. This approach ensures that highly correlated or redundant variables do not bias the model, while retaining those that meaningfully contribute to predicting *P. deleoni* distribution.

Additionally, a preliminary analysis assessed whether *P. deleoni* is in environmental equilibrium or pseudo-equilibrium with its surroundings. Environmental equilibrium refers to a state where the species occupies the full range of suitable conditions within its historically accessible area, implying no significant dispersal barriers or biotic constraints (López-Reyes et al. 2023). In contrast, pseudo-equilibrium indicates unoccupied, but suitable environments due to geographic barriers, biotic interactions or anthropogenic factors (López-Reyes et al. 2023). A species is considered to be in ecological imbalance when it is absent from climatically suitable areas or does not occupy all suitable environments or, as in our case, regions where both the climate and the availability of hosts are suitable. (Peterson et al. 2008, Barve et al. 2011, Qiao et al. 2015, López-Reyes et al. 2023). This is evaluated in environmental space, following Hutchinson’s duality (Colwell and Rangel 2009), where geographically close sites can differ in environmental conditions, leading to pseudo-equilibrium. These concepts are key for modelling species distributions and evaluating model robustness, especially when projecting beyond observed ranges (López-Reyes et al. 2023). Due to limited historical host records, we assumed the current distribution of *Juniperus* approximates past accessibility in Mexico. While a limitation, this provides a first approximation within the accessible area (“M”) framework. The “M” region (sensu Soberon and Peterson (2005)) was defined using the distribution of host plants (*J. flaccida*, *J. martinezii*, *J. poblana* and *J. deppeana*) as a proxy for long-term accessibility. This assumes current host ranges reflect historical availability and offers a realistic ecological basis for *P. deleoni*. A 40 km buffer, as used for host modelling, was applied to delineate “M”, accounting for the patchy nature of *Juniperus* forests and ensuring sufficient coverage for predictions.

To visualise the relationship between beetle occurrences and environmental conditions, a bivariate scatterplot was generated. The y-axis represented temperature seasonality, while the x-axis represented annual precipitation. Environmental values associated with the beetle's presence points were overlaid on to the environmental space defined by values extracted from points within the "M" buffer. This buffer was inferred, based on the geographic distribution of its confirmed and potential host plants: *Juniperus flaccida*, *J. deppeana*, *J. martinezii* and *J. poblana*. Should species records fall outside the environmental space represented within this historically accessible area, in that case, it may indicate that *P. deleoni* is not in equilibrium with its environment and is, instead, experiencing negative biotic interactions (e.g. competition, parasitism), range expansion or contraction or occupying a transitional or recently disturbed habitat. In such cases, the performance of ecological niche models and spatial distribution models may be reduced, resulting in lower predictive accuracy or increased uncertainty in model projections (López-Reyes et al. 2023).

The records of *Phloeosinus deleoni* were cleaned before modelling, applying the same criteria used for the records of its host species. Duplicate or spatially redundant records (overlapping records) were eliminated to avoid overestimating environmental suitability in the model, following methodological recommendations to minimise spatial bias in species distribution models (Boria et al. 2014). To establish the potential distribution of *P. deleoni*, a species distribution analysis was performed using the Maxent maximum entropy algorithm (Peterson 2003, Phillips et al. 2004, Phillips et al. 2006, Peterson et al. 2008), which is efficient for scarce data. The models were parameterised with different configurations to optimise their calibration using the Kuenm package (Cobos et al. 2019a) in RStudio version 4.2 (Rstudio 2021). Two regularisation multiplier values (0.1 and 2), two feature classes (linear and quadratic) and the predictor variables for each species were used (Cobos et al. 2019b, Nuñez‐Penichet et al. 2022). For evaluation and performance, the resulting models were assessed using the partial Receiver Operating Characteristic (ROC) curve test (Peterson et al. 2008).

Their predictive ability was analysed by considering the omission rate (E = 5%) and selection using the corrected Akaike Information Criterion (AICc) for small samples, prioritising statistically significant models or those with omission rates below 5% and AICc values < 2 (Warren and Seifert 2011). The one with the best omission and AICc parameters was selected from the resulting models. Once selected and calibrated, the model was parameterised in Maxent with two bootstrap replicates (Phillips et al. 2017, Nuñez‐Penichet et al. 2022). A Jackknife test was performed in Maxent to analyse the contribution of the selected variables. The analyses and results can be found in the GitHub repository (Github 2025). Finally, the maps were exported to ArcGIS for editing and visualisation.

## Results

**Figure 1.**
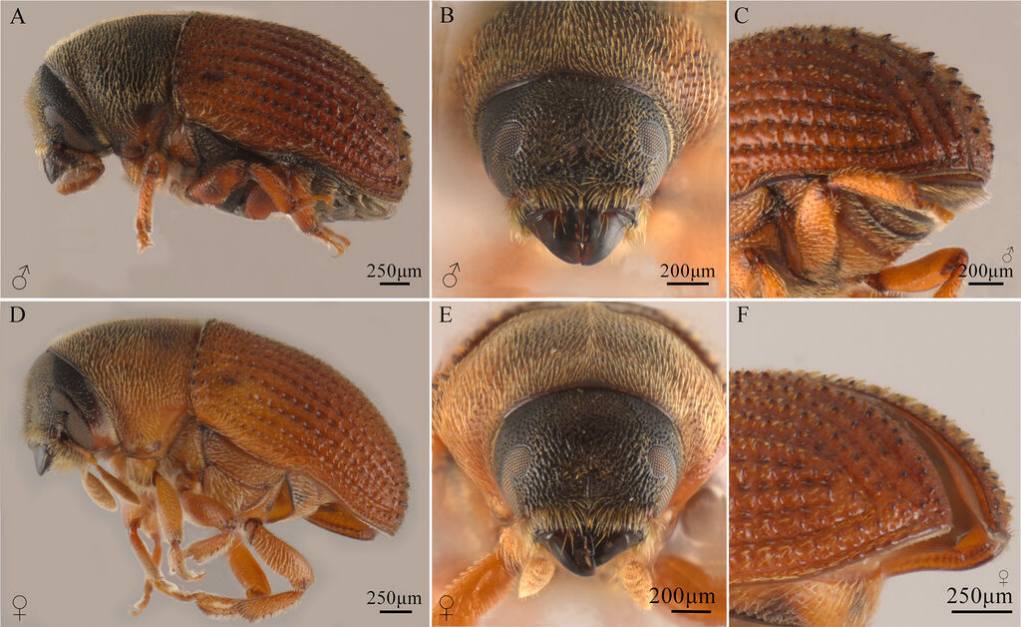
Male and female adults of *Phloeosinus deleoni*. **a** Male lateraldorsal view; **b** Male head; **c** Male elytral declivity; **d** Female lateraldorsal view; **e** Female head; **f** Female elytral declivity.

The records of *P. deleoni* found in entomological collections and bark beetle databases were corroborated through morphological identification using Wood’s identification keys (Wood 1992, Wood 2007) (Fig. 1). All examined adults displayed characters proposed for the species. Males (Fig. 1a–c) have a dark brown body and lighter-coloured elytra. The head has a broad, transversely impressed frons, convex dorsally and gradually ascending towards the epistomal margin ventrally (Fig. 1b). The eyes are large and elongate, separated from the frons by a sharply elevated medial carina extending from the epistomal margin to the upper level of the eyes. The antennae have an asymmetrically compressed club. The pronotum is 0.86 times as long as wide, with small, deep, closely-spaced punctures and abundant short pubescence. The elytral disc is entirely punctate, with scale-like setae becoming stouter and more prominent towards the declivity. The elytral slope is convex and gradual, beginning at the middle of the elytra. Interstriae I and III are weakly elevated, each armed with pointed teeth: interstria I bears three or four, while interstria III has seven to nine. Interstriae II are as wide as I and III, with about half of the males exhibiting a small tubercle near the apex. The vestiture is fine and hair-like, abundant near the epistoma, becoming coarser towards the declivity. Interstriae have fine crenulations, with punctures usually more pronounced along the anterior margins. Females (Fig. 1d–f) are similar to males, but have a more convex frons (Fig. 1e). Their interstriae exhibit larger and more abundant discal crenulations. The declivital teeth are slightly smaller, with interstriae I, II and III each bearing eight or nine pointed teeth of equal size (Fig. 1f).

In addition to these features, females exhibit several diagnostic characters newly proposed by Fernández-Campos et al. (2025), which provide further refinement for species identification: body length ranges from 2.7 to 3.3 mm; there are 13 crenulations per side on the margin of the elytral disc; five denticles are present on both interstriae I and III on the elytral declivity. The pronotum displays rounded lateral and anterior edges, with well-defined posterior angles and margins. The elytral declivity is slightly curved and begins in the second third of the elytra. Female sternite IX has a curved posterior margin, anterior margins that are slightly convex and rounded at the apices and a posterior width greater than its length.

A total of 22 distributional records of *Phloeosinus deleoni* were obtained from eight states in Mexico. These records include specimens collected for this study as well as those examined from biological collections — the oldest record dating back to 1953, while the most recent was collected in 2024. The species was mainly associated with *Juniperus flaccida* (11 records) and unidentified *Juniperus* species (10 records). In the recent collection in the State of Oaxaca, *P. deleoni* was documented and verified feeding on dying trees of *J. deppeana* (one record) (Table 1). *Phloeosinus deleoni* was found in different biogeographic provinces, including the Sierra Madre Occidental, Sierra Madre Oriental, the Transmexican Volcanic Belt, the Chihuahuan Desert, the Basin of the Balsas River and the Sierra Madre del Sur (Table 1). Notably, the record from Teotitlán del Valle, Oaxaca, represents the first documented occurrence of *P. deleoni* on *J. deppeana*, expanding the known host range of this species.

Out of the total records (22) listed in Table 1, ten (10) records from Oaxaca, Michoacán, Hidalgo, Jalisco, Durango and Chihuahua lack specific host identification. Consequently, these records were assigned to *Juniperus* sp. as the presumed host. In Oaxaca, *J. flaccida* and *J. deppeana* have co-distributed populations, making both potential hosts. Similarly, in Michoacán, populations of *J. deppeana*, *J. flaccida* and *J. poblana* occur closely, leading to uncertainty in host identification. The host records from Durango suggest sympatry amongst *J. flaccida*, *J. deppeana* and *J. poblana*, further complicating host determination. Lastly, the Chihuahua record is associated with nearby populations of *J. deppeana*. In terms of geographic expansion, new state-level records were obtained for Durango, where *P. deleoni* had not been previously reported. The records from Nuevo León and Oaxaca also provide additional confirmation of the species’ occurrence in these regions. The overall distribution of *P. deleoni* remains within previously defined limits.

**Figure 2.**
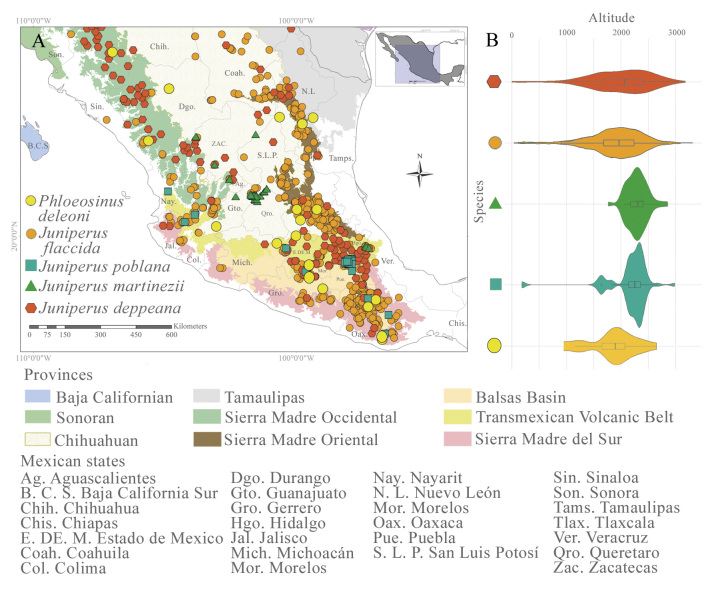
Geographic distribution of *Phloeosinus deleoni* and its host plants (*Juniperus flaccida*, *Juniperus poblana*, *Juniperus martinezii* and *Juniperus deppeana*) using the biogeographic provinces of Mexico. **A** Geographic distribution of *Phloeosinus deleoni* and its host plants, shown within the biogeographic provinces of Mexico; **B** Altitudinal ranges of *P. deleoni* and its host species.

The records of *P. deleoni* and *Juniperus* spp. (*J. flaccida*, *J. poblana*, *J. martinezii* and *J. deppeana*) are mainly distributed in the provinces of the Transmexican Volcanic Belt (TVBP) and the Sierra Madre del Sur (SMS) (Fig. 2). The highest number of records of *P. deleoni* (five records) are found in the provinces of the Sierra Madre Oriental (SMORP) (five records), Balsas Basin (BBP) (four records), the SMS (four records) and the Chihuahuan Desert (CDP) (four records) (Fig. 2). In the provinces of the Sierra Madre Occidental (SMOP) (three records) and the TVBP (two records), there were few records (Fig. 2). Host records follow the same pattern mentioned above, with *J. deppeana* having the highest number of records (793 records) in the SMOP, TVP and SMSP provinces. *Juniperus flaccida* displays the broadest distribution in the SMOP, TVBP and SMSP provinces. *Juniperus poblana* has a large population in the TVP in the northern part of Puebla, whereas *J. martinezii* has been recorded in the CDP in the northern part of Querétaro. Both *J. poblana* and *J. martinezii* have been recorded in the State of Aguascalientes (Fig. 2).

*Phloeosinus deleoni* occurs across an altitudinal range of 308 to 2652 m a.s.l., with a mean elevation of 1738 m. This range broadly overlaps with those of *Juniperus deppeana*, *J. poblana*, *J. flaccida* and *J. martinezii* (Fig. 2b). *Juniperus deppeana* is found between 308 and 2652 m a.s.l. (mean: 1734 m), sharing much of its elevational distribution with *J. poblana* (186–2980 m, mean: 2980 m) and, to a lesser extent, with *J. martinezii* (1751–2851 m, mean: 2289 m), as well as with *P. deleoni*. *Juniperus flaccida* spans a broader elevational range, from 100 to 3309 m a.s.l. (mean: 1918 m), partially overlapping with that of *P. deleoni*. *Juniperus poblana* also shows substantial altitudinal overlap with *J. martinezii* and, to a lesser degree, with *P. deleoni*, *J. deppeana* and *J. flaccida*. Finally, *J. martinezii* (1751–2851 m, mean: 2289 m) exhibits significant altitudinal overlap with both *J. deppeana* and *J. poblana* (Fig. 2b).

**Figure 3.**
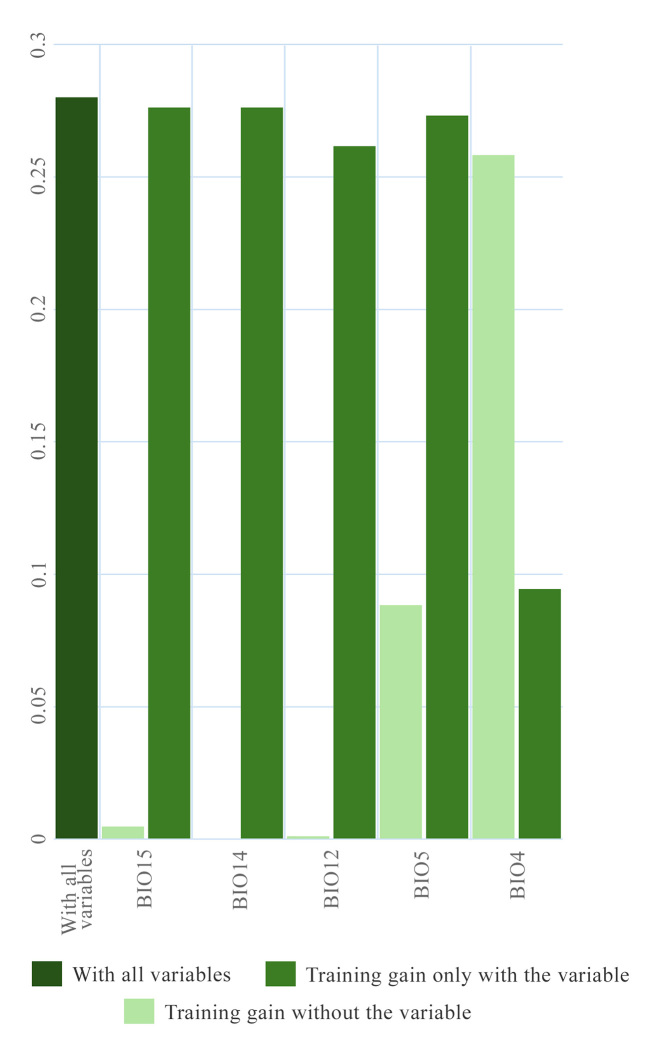
Histogram showing the relative contribution of the variables in the potential distribution model of the *Phloeosinus deleoni*.

The Jackknife test showed that the variables contributing to the model gain (Phillips et al. (2006) indicate a measure indicating how much the predictive model improves when a particular variable is included, helping to identify which factors are most important in predicting the distribution of the species) for *P. deleoni* were temperature seasonality (BIO4), the maximum temperature of the warmest month (BIO5), annual precipitation (BIO12), precipitation of the driest month (BIO14) and precipitation seasonality (BIO15). Of them, BIO4 was the environmental variable with the highest gain when used in isolation and the variable that decreased the gain when omitted (Fig. 3).

**Figure 4.**
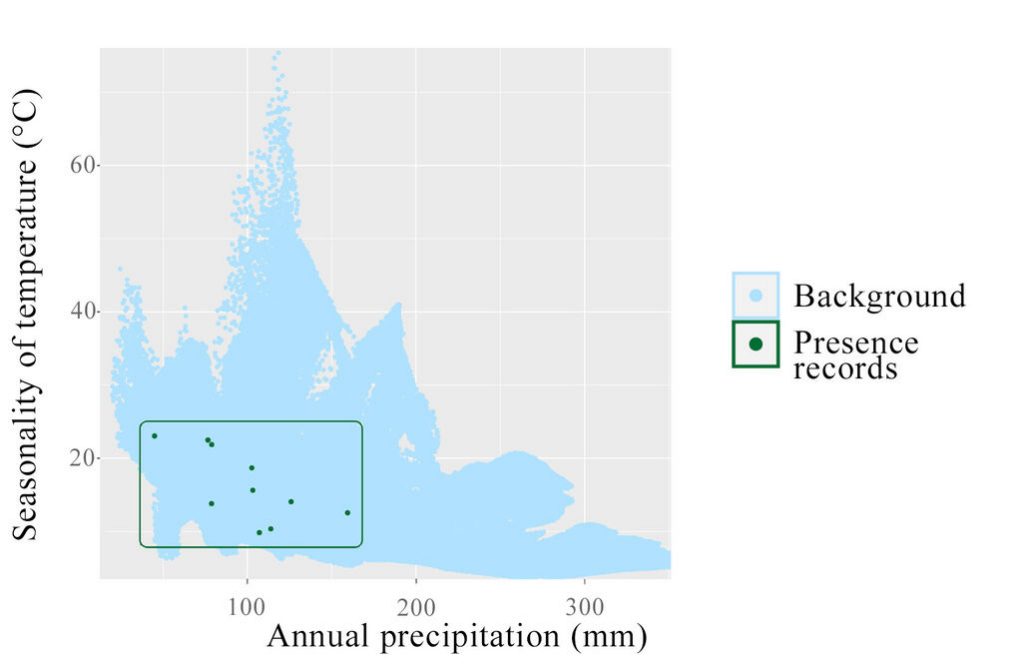
Environmental conditions within the historical accessibility area of *Phloeosinus deleoni*. The green dots represent presence records of the species. In contrast, the blue dots indicate the range of environmental conditions available within its historically accessible area (M region), inferred from the distribution of its confirmed and potential host plants (*Juniperus flaccida*, *J. deppeana*, *J. martinezii* and *J. poblana*).

The preliminary analysis to assess whether *Phloeosinus deleoni* is in environmental equilibrium or pseudo-equilibrium with its surroundings suggests that the species is currently in a state of pseudo-equilibrium. This is indicated by its failure to occupy all environmentally suitable conditions within its accessible area. The analysis focused on two key WorldClim variables identified as influential in the species distribution model: temperature seasonality (Bio4) and annual precipitation (Bio12). With temperature seasonality, *P. deleoni* has been recorded in locations with mean annual temperatures ranging from 8.9 to 32°C. Due to the lack of direct information on the species’ fundamental niche and considering that these values are derived from climate data extracted from occurrence records rather than from direct field measurements, this range should be interpreted cautiously as part of the realised niche, rather than as a definitive physiological or adaptive threshold. Similarly, precipitation data indicate that *P. deleoni* occurs in regions receiving between 49 and 157 mm of annual rainfall (Fig. 4). These findings support the hypothesis of pseudo-equilibrium because they show that *P. deleoni* occupies only a subset of the environmentally suitable conditions available within its accessible area. In other words, although the species is present in areas with suitable climate and host availability, it is absent from different regions that are also suitable according to the modelled environmental space. This pattern indicates that factors, such as dispersal limitations, biotic interactions or recent disturbances, may prevent the species from wholly occupying all suitable conditions, which is consistent with the concept of pseudo-equilibrium. Consequently, these results justify considering both the beetle and its host plants are in pseudo-equilibrium when interpreting potential distribution models, suggesting that the model is unlikely to overestimate or underestimate the species’ actual range substantially.

The evaluation model resulted in only one model that met the selection criteria: it was statistically significant. It met the omission rate, the partial Receiver Operating Characteristic (ROC) and the Akaike Information Criterion (AICc). The resulting model had a regularisation multiplier of two linear feature classes and a mean area under the curve (AUC) of 1.809. The omission rate was 0, the AICc was 244.7 and the delta AICc was 0.397.

**Figure 5.**
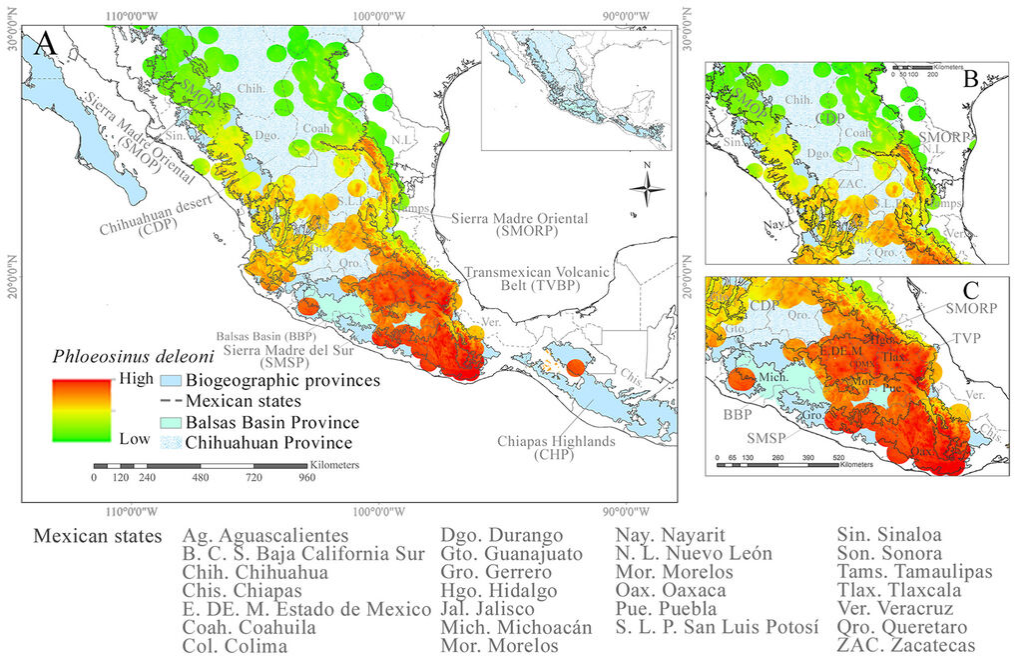
**A** Potential distribution of *Phloeosinus deleoni*; **B** Potential distribution in the Sierra Madre Oriental Province (SMORP); **C** Potential distribution in the Transmexican Volcanic Belt Province (TVBP). Abbreviations: BCP, Baja California Province; BBP, Balsas Basin Province; SMSP, Sierra Madre del Sur Province; CP, California Province; CHP, Chiapas Highlands Province; CDP, Chihuahuan Desert Province; SMOP, Sierra Madre Occidental Province; SMORP, Sierra Madre Oriental Province; TVBP, Transmexican Volcanic Belt Province.

The potential distribution model of *Phloeosinus deleoni* identified three highly suitable biogeographic provinces: Sierra Madre Oriental (SMOR), Transmexican Volcanic Belt (TVBP) and Sierra Madre del Sur (SMSP) (Fig. 5a). Additionally, smaller and more scattered areas of high and medium suitability were detected in the Sierra Madre Occidental (SMOP), Chihuahuan Desert (CDP) and Balsas Basin (BBP) (Fig. 5a, b). The highest suitability areas are concentrated in TVBP and SMSP (Fig. 5c). Within SMOP, medium-suitability areas are present in the southern portion, near the border with TVBP, where small patches of *Juniperus flaccida* forests occur. In the eastern TVBP, high-suitability regions were detected, whereas central and western TVBP also contain extensive high-suitability zones. In SMORP, highly suitable areas are primarily located in the southern region, with additional suitable patches in the northern mountainous areas. For SMSP, high environmental suitability extends across the western portion, with a larger continuous area in the east. In CDP, which borders SMOP and SMORP, medium-suitability zones are primarily located in the southern region (Fig. 5b). The BBP province contains scattered areas of high and medium suitability (Fig. 5c).

The potential distribution model of *Phloeosinus deleoni* indicates areas of medium suitability in the southern regions of Chihuahua and Durango, adjacent to a high-suitability zone in the eastern part of Jalisco (Fig. 5b). In the central and western portions of the study area, extensive high-suitability zones are identified in Estado de México, Morelos, Puebla and Tlaxcala (Fig. 5c). The southern regions of San Luis Potosí and Hidalgo also contain significant areas of high suitability (Fig. 5c). In the northern region, particularly in mountainous areas, highly suitable zones are detected in Nuevo León and Coahuila (Fig. 5b). At the same time, western Guerrero and eastern Oaxaca also exhibit high suitability for the species. Additionally, in the southern regions of Jalisco, Aguascalientes and Guanajuato, areas of medium suitability are present (Fig. 5).

## Discussion

This study delineated the distribution of *Phloeosinus deleoni* and modelled its potential distribution in Mexico, considering host availability and environmental variables as key factors. Our results support the hypothesis that *P. deleoni* occupies regions with ecological similarities to multiple *Juniperus* species, particularly in xeric and semi-arid environments (Schwarzbach and Adams 2006, Adams and Schwarzbach 2015, Adams et al. 2017, Adams et al. 2018). This is consistent with more recent phylogenomic hypotheses for *Phloeosinus*, which propose that *P. deleoni* is part of a specialised clade of species that feed on *Juniperus* (Fernández-Campos et al. 2025). The predicted distribution overlaps with the ranges of *J. flaccida*, *J. martinezii*, *J. poblana* and *J. deppeana*, reinforcing the strong association between the beetle and these *Juniperus* species (Wood 1982, Atkinson 2024). For the remaining *Juniperus* species, the lack of precise host identification in collection specimens and ongoing taxonomic uncertainties complicate the determination of the beetle’s complete host range (Schwarzbach and Adams 2006, Adams and Schwarzbach 2015). We emphasise that, while *P. deleoni* primarily uses *J. flaccida* (~ 90% of records), occasional utilisation of *J. deppeana* suggests some host flexibility, warranting further investigation (Evenden et al. 2014,Cervantes-Espinoza 2022, Fernández-Campos et al. 2025).

Twenty-two occurrence records were compiled from scientific collections, online databases, field collections and new records in Durango and Oaxaca. These confirm the presence of *P. deleoni* in the biogeographic provinces of the Sierra Madre del Sur, Sierra Madre Oriental, the Sierra Madre Occidental, the Transmexican Volcanic Belt, the Chihuahua Desert and the Balsas Basin, refining previous records in Chihuahua, Durango, Hidalgo, Jalisco, Michoacán, Morelos, Nuevo León and Oaxaca (Wood 1982, Atkinson and Equihua 1985, Bright and Skidmore 1997). The collection made in Teotitlán del Valle, Oaxaca, provides the second confirmed record of *J. deppeana*, supporting the hypothesis that *P. deleoni* has a broader range of hosts than previously documented beyond flexible-leaved junipers, such as *J. flaccida*, *J. poblana* and *J. martinezii* (Schwarzbach et al. 2006, Schwarzbach and Adams 2006, Adams 2014, Adams and Schwarzbach 2015).

The distribution models identified three major regions of high environmental suitability: Sierra Madre del Sur, Sierra Madre Oriental and the Transmexican Volcanic Belt. These areas, primarily pine-oak and pine-juniper forests at low to mid-elevations, combine moderate to high temperatures with semi-arid conditions (Wood 1986, Salinas-Moreno et al. 2010, Smith et al. 2010, Aguilar-Soto et al. 2015, González-Martínez 2021). The overlap between suitable climate and *Juniperus* distribution highlights the joint role of biotic and abiotic factors, consistent with patterns observed in other phloeophagous beetles (Salinas-Moreno et al. 2010, Peterson et al. 2011).

*Phloeosinus deleoni* exhibits host flexibility, particularly using *J. deppeana*, a species phylogenetically distant from the flexible-leaved *Juniperus.* This aligns with the concept of “flexible co-specialisation”, where beetles show strong preferences, yet retain the ability to colonise alternative hosts (Glasser 1982, Yotoko et al. 2005, Uckele et al. 2021, see also Cognato et al. (2021) and Takagi (2022)). This interpretation is supported by the predominance of records on *J. flaccida* (~ 90%), coupled with confirmed occurrences on *J. deppeana* (Wood 1982, Schwarzbach and Adams 2006, Adams 2014). Nearly half of the available records (10 of 22, 45%) lack host information, limiting resolution of host–beetle associations (Adams 2014, Cognato et al. 2021, Du et al. 2022).

The co-existence with multiple species of *Juniperus*, together with the colonisation of phylogenetically distant hosts, suggests that *Phloeosinus deleoni* may have ecological flexibility that allows *P. deleoni* to exploit a broader range of hosts (Vasek 1966, Rawat and Everson 2012, Herrerías Mier and Nieto de Pascual 2020). The altitudinal range of phytophagous insects is usually parallel to that of their host plants and is determined by host availability, environmental tolerance, degree of specialisation and biotic interactions (Hodkinson 2007, Futuyma and Agrawal 2009, Gaston and Fuller 2009). The altitudinal overlap with all *Juniperus* species analysed indicates that its colonisation strategy is flexible, rather than strictly generalist, allowing it to persist in diverse environmental conditions and follow changes in host distribution induced by environmental change (Méndez-Encina et al. 2021).

The preliminary analysis of environmental equilibrium indicates that *P. deleoni* does not fully occupy all climatically suitable habitats (Bentz et al. 2019, Qiao et al. 2015, López-Reyes et al. 2023). This pseudo-equilibrium suggests that its current distribution may lag behind available habitat due to host dynamics or dispersal constraints (McCain and Garfinkel 2021, Lalechère et al. 2025). Consequently, undocumented associations with *J. martinezii* and *J. poblana* remain possible (Blackman 1942, Adams 2004). Future research should focus on studying areas with *J. martinezii* to verify the presence of *P. deleoni* and further investigate its associations with hosts. In addition, experimental studies on host selection and chemical attraction would be valuable for understanding the role of volatile compounds in the ecology of *P. deleoni*.

Environmental variables played a key role in modelling the distribution. Temperature of the warmest month (Bio4) and annual precipitation (Bio12) were the main contributors to model gain, as identified by the Jackknife test. High temperatures and low precipitation, typical of dry seasons, are associated with increased tree mortality and accelerated bark beetle development (Faccoli 2009, Creeden et al. 2014, González-Hernández et al. 2018, Bentz et al. 2019, Carrillo-Aguilar et al. 2021). As an early-stage saproxylic beetle, *P. deleoni* colonises recently dead or weakened trees (Blackman 1942), making it sensitive to climatic conditions that influence host vulnerability. These findings demonstrate the predictive power of ecological niche models when biologically significant variables are incorporated (Araújo et al. 2019, Cobos et al. 2019b).

In parallel, chemical ecology provides an additional explanatory layer. Bark beetles often respond to volatile compounds such as α-pinene, linalool and camphor, which are abundant in *Juniperus* (Chararas et al. 1981, Adams et al. 1984, Adams and Schwarzbach 2015, Krokene 2015). Although *P. deleoni* has not been specifically studied, related species rely on these compounds for host selection (Chararas et al. 1981, Krokene 2015). Moreover, the degradation of secondary metabolites in weakened or dead trees can reduce host defences, potentially broadening host availability for colonisation (Lindgren and Raffa 2013, Hulcr et al. 2015, Johnson et al. 2018, Cognato et al. 2021). Together, these environmental and chemical cues contribute to explaining not only the beetle’s distribution patterns, but also its ecological strategy.

Biogeographically, *P. deleoni* is absent from the Chiapas Highlands, unlike *Phloeosinus tacubayae* Hopkins, 1905, which also occurs in cooler and more humid Neotropical areas (Wood 1982, Cervantes-Espinoza 2022). This restriction suggests that the Balsas Basin may act as a barrier between Sierra Madre del Sur and the Transmexican Volcanic Belt, while mountain systems and transitional forests could function as dispersal corridors (Halffter 1987, Halffter and Morrone 2017, Morrone 2020). The distribution of *P. deleoni* resembles that of *Dendroctonus mexicanus* Hopkins, 1909, which is similarly concentrated in the Transmexican Volcanic Belt (González-Hernández et al. 2018).

Altogether, the convergence of climate suitability, host distribution and forest composition suggests that *P. deleoni* is not strictly monophagous, but rather exhibits partial specialisation with ecological flexibility (Aguilar-Soto et al. 2015, González-Martínez 2021). Its distribution aligns with the Mexican Transition Zone (MTZ), a biogeographic hotspot where Nearctic taxa expanded during the Miocene–Pleistocene (Halffter 1987, Halffter 2017, Halffter and Morrone 2017, Morrone 2020, Cervantes-Espinoza et al. 2022). Similar diversification processes have been documented in *Juniperus*, as well as in vertebrates such as *Peromyscus* Gloger, 1841 and *Sceloporus* Wiegmann, 1828 (Halffter and Morrone 2017). Thus, *P. deleoni* provides a model system for understanding how climate, host range and biogeographic history jointly shape insect distributions in temperate montane and semi-arid environments of central and southern Mexico.

## Conclusion

This study delineated the potential distribution of *Phloeosinus deleoni* in Mexico using spatial distribution models that integrated occurrence records, host plant availability and climatic variables. The results confirm a close association with *Juniperus flaccida* and *J. deppeana* and provide field-based evidence of a second direct feeding record on *J. deppeana*, expanding current knowledge of the species’ host range. The most influential climatic variables were the temperature of the warmest month and annual precipitation, which, together with host distribution, explain the presence of the species in temperate and semi-arid regions of the Mexican Transition Zone. Although climatically suitable areas exist where *P. deleoni* has not been recorded, this discrepancy is likely driven by local host dynamics, limited dispersal capacity or geographic barriers. Taken together, our findings re-affirm that both biotic and abiotic factors shape its current distribution and provide a solid foundation for future research on its ecology, genetic structure, adaptive potential and responses to environmental change within the Mexican Transition Zone.

## Figures and Tables

**Table 1.** Collection records and field surveys of *Phloeosinus deleoni* in Mexico.

**State**	**Locality**	**Longitude (N), Latitude (W)**	**Altitude (m a.s.l.)**	**Biogeographic Province**	**Host**	**Date**	**Collection/Vaucher**
Chihuahua	Huachochi	27°51'00", -107°30'00"	2400	Sierra Madre Occidental	Juniperus sp.	29-IV-1981	UACC-14762
Durango	Mexiquillo	24°06'95", -104°36'50"	1900	Sierra Madre Occidental	Juniperus sp.	14-VIII-1982	IPN-PD
Durango	Mexiquillo	24°06'95", -104°36'50"	1900	Sierra Madre Occidental	Juniperus sp.	14-VIII-1983	IPN-PD
Durango	Pueblo Nuevo	23°43'37", -105°28'37"	2080	Chihuahuan Desert	Juniperus sp.	7-XI-1964	IPN-PD
Hidalgo	Cardonal	20°37'36", -99°06'50"	2250	Sierra Madre Oriental	Juniperus flaccida	27-lll-1981	CEAM-3687
Hidalgo	Jacala	21°01'01", -99°10'58"	1928	Sierra Madre Oriental	Juniperus flaccida	-	USNM-29320
Hidalgo	Jacala	21°01'01", -99°10'58"	1928	Sierra Madre Oriental	Juniperus flaccida	VI-1953	USNM-37455
Hidalgo	Zimapan	21°41'01", -99°15'13"	1340	Chihuahuan Desert	Juniperus sp.	11-VII-1967	USNM-37454
Jalisco	Guadalajara	20°40'22", -103°21'03"	1869	Chihuahuan Desert	Juniperus sp.	22/IV/1977	USNM-29321
Michoacán	Tuxpan, Carr. Toluca-Morelia	19°37'43", -100°28'44"	2418	Transmexican Volcanic Belt	Juniperus sp.	-	Unknown-Wood-29322
Michoacán	Cd. Hidalgo	19°40'59.9", -100°34'01.2"	2652	Transmexican Volcanic Belt	Juniperus flaccida	30-X-1980	CEAM-3688
Morelos	Cuernavaca	18°58'24", -99°15'30"	1850	Basin Balsas	Juniperus flaccida	19-VIII-1981	IPN-PD
Morelos	Tepoztlán	18°58'51.6", -99°11'31.2"	1640	Basin Balsas	Juniperus flaccida	15/I/1982	CEAM-3691
Morelos	Cuernavaca	18°58'00.1", -99°15'00.0"	1450	Basin Balsas	Juniperus sp.	19-VII-1981	CEAM-3690
Morelos	Cuernavaca	18°58'00.1", -99°15'00.0"	1450	Basin Balsas	Juniperus sp.	21-IX-1982	CEAM-129699
Nuevo León	Galeana	24°49'31", -100°04'38"	1640	Chihuahuan Desert	Juniperus flaccida	11/XI/2018	CNIN-PD01
Nuevo León	Iturbide	24°43'16", -99°53'46"	1440	Sierra Madre Oriental	Juniperus flaccida	14-XI-2018	CNIN-PD02
Nuevo León	Iturbide	24°43'30.0", -99°54'21.6"	1440	Sierra Madre Oriental	Juniperus flaccida	9-XI-1984	CEAM-129700
Oaxaca	San Francisco Telixtlahuaca	17°22'34.3", -96°54'58.0"	1750	Sierra Madre del Sur	Juniperus flaccida	22-X-2011	UTIC-35758
Oaxaca	San Mateo Río Hondo	16°11'04.2", -96°30'43.6"	2000	Sierra Madre del Sur	Juniperus sp.	11-V-1977	Unknown-Wood
Oaxaca	San Baltazar Guelavila	16°47'09.6", -96°19'44.0"	1801	Sierra Madre del Sur	Juniperus flaccida	19-X-2011	MSUC-35759
Oaxaca	Teotitlán del Valle	17°27'50.1", -97°15'14.1"	2065	Sierra Madre del Sur	Juniperus deppeana	3-VI-2024	CNIN-PD20
